# Enhancing the Electrochemical Performance of Ni-Rich LiNi_0.88_Co_0.09_Al_0.03_O_2_ Cathodes through Tungsten-Doping for Lithium-Ion Batteries

**DOI:** 10.3390/nano12050729

**Published:** 2022-02-22

**Authors:** Rui Zhang, Hengrui Qiu, Youxiang Zhang

**Affiliations:** College of Chemistry and Molecular Sciences, Wuhan University, Wuhan 430072, China; zhangrui@whu.edu.cn (R.Z.); 2020102030003@whu.edu.cn (H.Q.)

**Keywords:** tungsten doping, phase transition, structural stability, electrochemical performance, LiNi_0.88_Co_0.09_Al_0.03_O_2_

## Abstract

The tungsten-doped (0.5 and 1.0 mol%) LiNi_0.88_Co_0.09_Al_0.03_O_2_ (NCA) cathode materials are manufactured to systematically examine the stabilizing effect of W-doping. The 1.0 mol% W-doped LiNi_0.88_Co_0.09_Al_0.03_O_2_ (W1.0-NCA) cathodes deliver 173.5 mAh g^−1^ even after 100 cycles at 1 C, which is 95.2% of the initial capacity. While the capacity retention of NCA cathodes cycled in identical conditions is 86.3%. The optimal performances of the W1.0-NCA could be ascribed to the suppression of impendence increase and the decrease in anisotropic volume change, as well as preventing the collapse of structures during cycling. These findings demonstrate that the W-doping considerably enhances the electrochemical performance of NCA, which has potential applications in the development of Ni-rich layered cathode materials that can display high capacity with superior cycling stability.

## 1. Introduction

Lithium-ion batteries (LIB), the popularity of which is growing rapidly, are used primarily in all kinds of electronic equipment. Currently, they are most often used in laptops, mobile phones, digital cameras, and other portable devices, as well as in electric and hybrid cars [1,2,3,4,5]. Among the commonly available LIB cathode materials, Ni-rich layered cathode materials, particularly LiNi_x_Co_y_TM_1−x−y_O_2_, are believed to be the best choice of power sources for the current electric vehicles [6,7,8,9,10,11,12,13]. The LiNi_0.8_Co_0.15_Al_0.05_O_2_ cathodes, for example, have been successfully applied to LIB to store electricity for Tesla electric vehicles, but it still has a cruising distance problem and insufficient cycle life [5,14,15]. It should be emphasized that the current strategy of achieving high energy densities in Ni-rich layered cathodes was to increase the nickel content, resulting in the development of LiNi_0.81_Co_0.15_Al_0.04_O_2_, LiNi_0.87_Co_0.1_Al_0.03_O_2_, and LiNi_0.88_Co_0.09_Al_0.03_O_2_ [16,17,18,19].

Although Ni-rich cathode materials have advantages in the high energy density with low material costs, they have greatly reduced the cycling performance with poor thermal stabilities and serious anisotropic volume change due to the deleterious H2-H3 phase (hexagonal to another hexagonal phase) transition, which hinders their commercialization [8,20,21,22]. The harmful H2-H3 phase transition generates internal microcracks in cathode particles because the H3 phase has a smaller unit cell volume [23,24]. To make matters worse, the issue of microcracking during cycling becomes more severe with increasing nickel content in the LiNi_x_Co_y_Mn_1−x−y_O_2_ (0.6 ≤ x ≤ 0.95) series, reported by Ryu et al. [25], and the cracking problem were also observed in the study of LiNi_x_Co_y_Al_1−x−y_O_2_ (x = 0.8, 0.88, 0.95) materials by Nam et al. [26]. In addition, the practical capacity of those commercial layered cathode materials with their nickel content near 80% has been limited to around 180–200 mAh g^−1^ to enhance cycle stability. And the comparatively high cobalt percentage is becoming increasingly problematic due to the soaring price of cobalt [3,15,27,28,29]. Therefore, it is a challenging task to develop Ni-rich cathodes with acceptable cycle stability.

Numerous strategies have been employed to strengthen the host structure by doping, coating, and establishing special architectures such as the core-shell model [9,13,27,30]. Atomic doping is widely considered to enjoy a promising development, with simple operation to significantly improve battery lifetime, rate capability, and structural integrity of nickel-rich cathode materials. In particular, atomic doping enhances the structural stability of nickel-rich cathode materials by adjusting the fundamental physicochemical properties of the materials at the crystal level, such as metal-oxygen covalency, phase transformation, charge redistribution, and lattice parameters [15,31]. Recently, researchers have been attempting to improve the structural stability of nickel-rich cathodes via replacing transition metals with atoms of different elements. According to reports, various atoms (Na, Al, Ti, Mn, Y, Zr, Gd, F, and B) have been used to dope the nickel-rich layered cathode materials, which led to a great improvement in the electrochemical performance of the materials [10,13,19,32,33,34,35,36]. Furthermore, Sun et al. have demonstrated that W-doping can surmount the problem in LiNiO_2_ with inherent structure instability and significantly improve its cycle performance and thermal properties without reducing its capacity [37].

Hence, we introduced W in Ni-rich layered LiNi_0.88_Co_0.09_Al_0.03_O_2_ cathodes via a simple doping process to considerably improve its cycling stability by overcoming inherent structural instability. Different quantities of W (0.5 and 1.0 mol%) are doped into pristine LiNi_0.88_Co_0.09_Al_0.03_O_2_ (denoted as NCA) cathodes. A comparative study of the morphology variations and the resulting cell performance of NCA, 0.5 mol% W-doped LiNi_0.88_Co_0.09_Al_0.03_O_2_ (W0.5-NCA), and 1.0 mol% W-doped LiNi_0.88_Co_0.09_Al_0.03_O_2_ (W1.0-NCA) cathode materials was performed to evaluate their electrochemical performances, structural stabilities, and microcracking properties. Facts have proved that an appropriate W-doping level (1.0 mol%) is expected to significantly enhance structural integrity, cycle reversibility, and rate performance, which opens up new possibilities for LIB with high energy densities.

## 2. Materials and Methods

### 2.1. Chemicals

Nickel sulfate hexahydrate was manufactured by Shanghai Lingfeng Chemical Reagent Co., Ltd. (Shanghai, China). Aluminum hydroxide, Cobalt (II) sulfate heptahydrate, Lithium hydroxide monohydrate, Ammonia solution, and Sodium hydroxide were supplied by Sinopharm Chemical Reagent Co., Ltd. (Shanghai, China). Tungsten trioxide was generated by Aladdin Industrial Co. (Shanghai, China).

### 2.2. Synthesis Procedure

**Figure 1:**
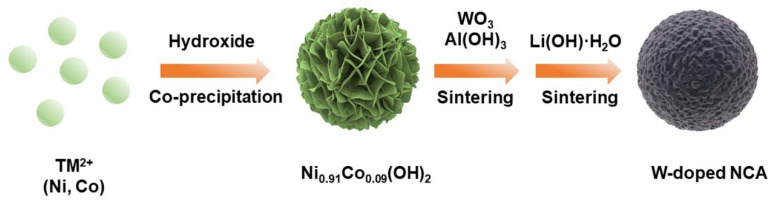
Schematic illustration of the preparation of W-doped NCA cathodes.

The synthesis of W doped NCA, which involves two steps: co-precipitation and annealing, is well illustrated in Figure 1. The pristine spherical Ni_0.91_Co_0.09_(OH)_2_ precursors were fabricated through a common co-precipitation method. NiSO_4_·6H_2_O and CoSO_4_·7H_2_O were dissolved in deionized water in required stoichiometric proportions (in order to obtain a transparent solution of 2.0 mol L^−1^). The mixed solution was injected into a 5 L tank reactor (with continuous agitation at the appropriate flow rate). Meanwhile, NH_3_·H_2_O solution (2 mol L^−1^) as chelating agents and NaOH solution (6 mol L^−1^) as precipitants were separately transferred to the tank reactor. The pH was almost maintained at 10.6. During the entire synthesis process, feeding speed, stirring speed, and the reaction temperature were well controlled. Finally, the collected Ni_0.91_Co_0.09_(OH)_2_ precursor powder was cleaned several times by deionized water, then dried at 100 °C for 12 h.

The prepared Ni_0.91_Co_0.09_(OH)_2_ precursor was mixed with a stoichiometric amount of Al(OH)_3_. After that, the mixture was calcined at 500 °C in an oxygen atmosphere for 6 h to synthesize the NCA precursor. The NCA was fabricated through a high-temperature lithiation process. The prepared NCA precursor was mixed with LiOH·H_2_O in a (Ni+Co+Al): Li mole ratio of 1:1.05. Then, the mixture was sintered at 730 °C for 12 h with flowing oxygen gas.

The obtained Ni_0.91_Co_0.09_(OH)_2_ precursor were homogeneously mixed with Al(OH)_3_ and WO_3_, ((Ni+Co+Al):W = 1:x (x = 0.5%, 1.0%) molar ratio) and calcined with the same procedure as above. While working to improve the electrochemical performance of Ni-rich LiNi_0.88_Co_0.09_Al_0.03_O_2_ cathodes, the W-doped NCA cathodes were prepared according to this analogous procedure.

### 2.3. Characterization

Powder X-ray diffraction (XRD) for the determination of the structure of the crystalline phases was characterized in the range of 2θ 10–80°, the scanning rate of 2°/min (by the Bruker D8 Advance powder diffractometer with Cu Kα radiation, λ = 1.5406 Å). Rietveld refinement was applied to refine structural parameters. The scanning electron microscopy (SEM) was conducted on a field emission scan electron microscope (Zeiss, SIGMA, Oberkochen, Germany). EDS energy dispersive X-ray spectrometer (Oxford Instruments, UltimMax 40, Oxford, UK) equipped on the scanning electron microscopy was used to visualize the spatial distribution of major elements for NCA and W-doping cathodes. The X-ray photoelectron spectroscopy (XPS, Thermo Fisher Scientific, Escalab 250Xi, Waltham, MA, USA) measurements were performed to perceive the state of relative elements.

### 2.4. Electrochemical Measurements

A coin cell (CR2016 with lithium metal disks as counter electrodes) was used to conduct electrochemical characterizations. The active material, polyvinylidene fluoride (PVDF) in N-methyl-2-pyrrolidone (NMP) and acetylene black in the weight ratio of 80:5:15 were taken and ground manually with a mortar and pestle. During the grinding process, the viscosity of the slurry was adjusted by adding an appropriate amount of NMP. The slurry was pasted on the aluminum foil, using the doctor blade gap of 100 microns. Then, it was dried in a vacuum at 100 °C for 24 h. The separator was Celgard 2300 microporous film. The electrolyte consisted of 1.0 M LiPF_6_ in ethylene carbonate (EC)/dimethyl carbonate (DMC) (1:1 by volume) solvents. The cells were assembled in a glovebox filled with high-purity argon gas. The galvanostatic charge-discharge test was performed using the Neware battery test system (Neware, CT-4008T, Shenzhen, China) with a voltage range of 2.8–4.3 V. Cyclic voltammetry (CV) was performed at a scan rate of 1.0 mV s^−1^ from 2.8–4.3 V. Electrochemical impedance spectroscopy (EIS) with an amplitude of 5 mV was obtained in the frequency range from 100 kHz to 0.01 Hz. CV and EIS were performed using the electrochemistry workstations (Shanghai CH instruments, CHI760C, Shanghai, China).

## 3. Results and Discussion

The SEM images in Figure S1 show the precursor Ni_0.91_Co_0.09_(OH)_2_ particles with a diameter of 5.0–6.5 μm. These spherical particles consist of nanosheets resembling flower petals. SEM elemental mapping is carried out for the precursor, shown in Figure S1c, which proves that both Ni and Co elements have a homogeneous distribution, and their element signals also overlap well with the O region. The structure of the precursor was performed by XRD, shown in Figure S2. Major X-ray diffraction reflections can be indexed as Ni(OH)_2_ (JCPDS: No. 14-0117).

**Figure 2:**
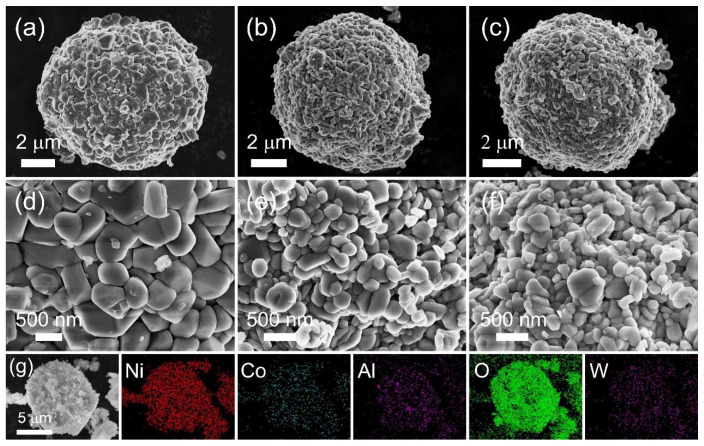
SEM images (**a**,**d**) NCA, (**b**,**e**) W0.5-NCA, (**c**,**f**) W1.0-NCA cathode materials. (**g**) Element mapping of Ni, Co, Al, O, and W for the cross-sectional of W1.0-NCA cathodes particle.

Figure 2 displays SEM images of the NCA and W-doped NCA cathodes. Although W was added in the Ni_0.91_Co_0.09_(OH)_2_ precursor prior to lithiation, W-doping had almost no effect on the morphology of the secondary particles. The average diameter of the secondary particles for NCA and W-doped NCA cathodes is about 8.5 μm. Notably, the SEM images in Figure 2d–f reveal that the size of the primary particles constituting the secondary particles tends to decrease as the content of W increases [38]. It might have resulted from W changing the surface energy of primary particles in the process of crystal growth [39]. The corresponding EDS mapping for the cross-sectional of W1.0-NCA cathodes particle is displayed in Figure 2g. It demonstrates that the element of W is uniformly distributed over the whole particle, similar to the distribution of other major components.

**Figure 3:**
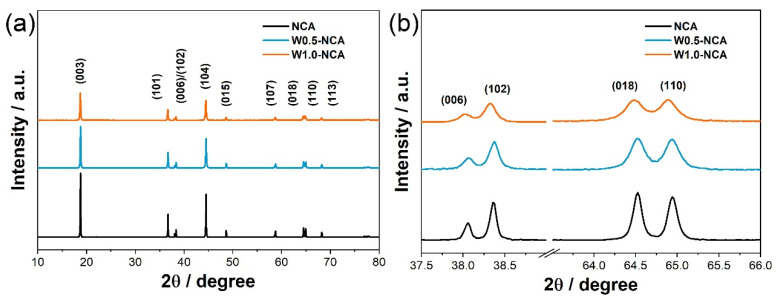
(**a**) XRD patterns of NCA, W0.5-NCA, W1.0-NCA, (**b**) the magnification region of 37.5–66.0°.

XRD patterns of the NCA and W-doped NCA cathodes are displayed in Figure 3a. It indicates that both NCA and W-doped NCA have a hexagonal α-NaFeO_2_-type layered structure with R$\bar{3}$m space group. No impurity phases can be observed, indicating that the W atoms are well inserted into the lattice of the NCA. The (006)/(102) and (108)/(110) reflections with clear peaks splitting in Figure 3b, demonstrate that the W-doping does not change the layered structure with high crystallinity of the cathodes. Rietveld refinement is conducted, and the lattice parameters of NCA, W0.5-NCA, and W1.0-NCA are presented in the appropriate Table 1 and Figure S3. During the Rietveld refinement of XRD patterns, the molecular models of W0.5-NCA and W1.0-NCA are assumed to LiNi_0.875_Co_0.09_Al_0.03_W_0.005_O_2_ and LiNi_0.87_Co_0.09_Al_0.03_W_0.01_O_2_, respectively. The (003)/(104) peak intensity ratio (I_(003)_/I_(104)_) decreases from 1.62 for NCA to 1.49 for W0.5-NCA, and 1.38 for W1.0-NCA [37,40], in agreement with the increase in cation mixing value (3.09 for NCA, 4.14 for W0.5-NCA, and 6.36 for W1.0-NCA). The specific value of I_(003)_/I_(104)_ decreased upon W dopant increasing, which may be due to the increase in Ni^2+^ to maintain the charge neutrality of cathode materials [38]. The c/a values of cathode materials are bigger than that of the ideal cubic dense packing structure (4.899), suggesting that W-doping does not affect the layered structure of the materials. Remarkably, W-doped NCA exhibits a larger unit cell volume than that of pristine NCA cathodes. The expansion of unit cells benefits the migration of Li^+^ and is conducive to rate performance [41]. In addition, the magnified XRD patterns in Figure 3b shows full width at half maxima broadening, the magnitude of which is proportional to the W content. This suggests that the W-doping of the NCA cathodes decreases the size of the primary particles as the W fraction increases. This result is very consistent with that shown by the SEM images in Figure 1.

**Figure 4:**
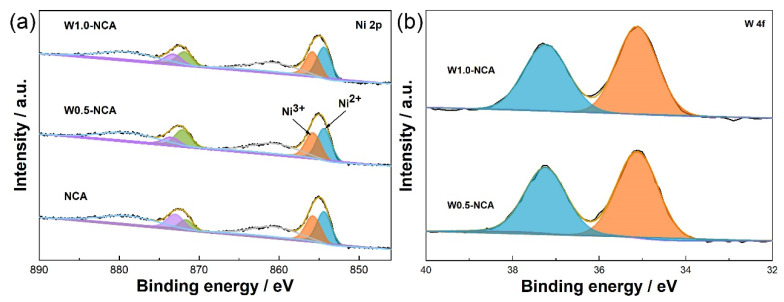
XPS spectra of (**a**) Ni 2p, (**b**) W 4f for NCA, W0.5-NCA, W1.0-NCA cathodes.

XPS measurements were performed to study the valence states of main elements, and all spectra were standardized with the C1s peak at 284.8 eV. In Figure S4, the major binding energy peaks of C, O, Al, Co, Ni, and W were all detected in NCA and W-doped NCA cathodes, while the binding energy peaks of W were observed in W-doped NCA cathodes. The Ni 2p XPS spectrum in Figure 4a exhibits two highlighted peaks at 854.9 and 872.6 eV, which correspond to Ni 2p_2/3_ and Ni 2p_1/2_, respectively. And their associated satellite peaks are at 861.3 and 879.5 eV. The Ni 2p_2/3_ spectra can be fitted to two peaks, one peak at around 854.4 eV assigned to Ni^2+^, the second peak at around 855.8 eV assigned to Ni^3+^. According to the simulation results of Ni 2p_3/2_ peak, the area ratio of Ni^3+^/(Ni^2+^ + Ni^3+^) of NCA, W0.5-NCA, and W1.0-NCA are 47.4%, 45.4%, and 43.9%, respectively. The result is in correspondence with the XRD analysis above. What is more, Figure 4b shows the original peak of W 4f and its fitting peak. It can be seen that the binding energy of W 4f_7/2_ and W 4f_5/2_ are respectively located at 35.1 and 37.2 eV, which confirms the existence of W^6+^ [15].

**Figure 5:**
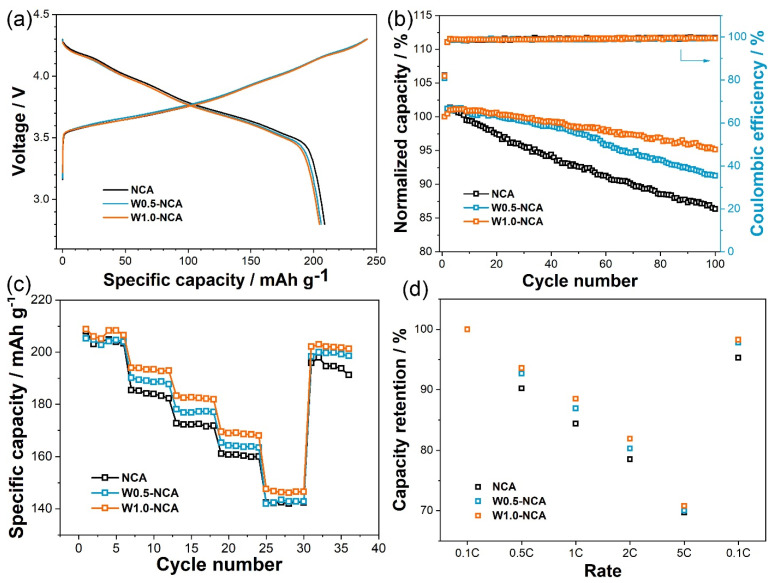
(**a**) Initial charge/discharge profiles at 0.1 C, (**b**) Cycling performance and coulombic efficiency at 1 C, (**c**) Rate capability of 0.1–5.0 C, and (**d**) Relative discharge capacity retention as a function of C-rate for NCA, W0.5-NCA, and W1.0-NCA cathodes between 2.8–4.3 V.

Figure 5a displays the initial charge-discharge profiles of NCA and W-doped NCA cathodes (W0.5-NCA, W1.0-NCA) at a rate of 0.1 C (20 mA g^−1^). The initial discharge specific capacities of NCA, W0.5-NCA, and W1.0-NCA, are 208.8, 206.0, and 204.6 mAh g^−1^, corresponding to the coulombic efficiencies of 86.2%, 85.1%, and 84.3%, respectively. The charge-discharge curves of all electrodes with almost the same characteristics from 2.8–4.3 V indicate that W-doping would not change the electrochemical properties of the cathode materials. Figure 5b reveals the cycling performance and coulombic efficiency conducted at a current density of 1.0 C. The NCA shows a volume attenuation, decaying from 182.4 to 157.5 mAh g^−1^, with capacity retention of 86.3% after 100 cycles. The cycling stability of NCA cathode materials is significantly improved by W-doping. W0.5-NCA and W1.0-NCA provide capacity retention values of 91.3% (185.6 to 169.4 mAh g^−1^) and 95.2% (182.3 to 173.5 mAh g^−1^), respectively. In addition, their coulombic efficiency increases to more than 98% after one cycle, and these values rise to 99% after 55 cycles. It exhibits the typical electrochemical properties of layered cathode materials. These results indicate that W1.0-NCA exhibits the best cycle stability. Furthermore, the rate capability is also tested of 0.1–5 C at 2.8–4.3 V, shown in Figure 5c,d. It is obvious that the discharge specific capacity of the three cathodes gradually decreases when the current increases. Compared to NCA, the W1.0-NCA shows distinctly improved rate performance. For example, W1.0-NCA exhibits a reversible specific capacity of 146.2 mAh g^−1^ at 5 C, which is 70.8% of its capacity at 0.1 C (206.5 mAh g^−1^). The capacity of NCA is 142.3 mAh g^−1^ at 5 C, which is 69.7% of its value at 0.1 C (204.3 mAh g^−1^). The improved rate performance is primarily related to the slight expansion of the lattice structure by W-doping, which results in the increase in the distance between the lithium layers and the rapid deintercalation of Li^+^ from the layered structures [41]. Combining the results of cycling and rate measurements, it is considered that the appropriate W dopant (1.0 mol%) could better enhance the electrochemical performances of the cathodes compared to others.

**Figure 6:**
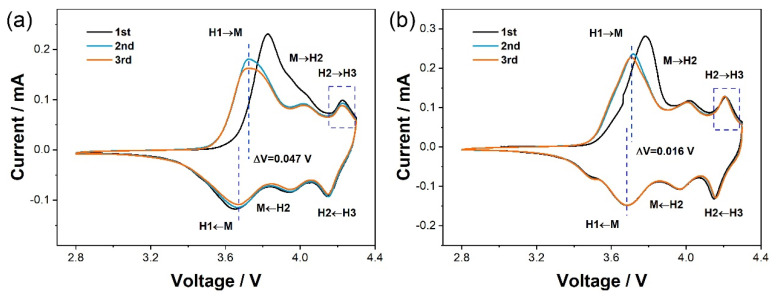
CV of (**a**) NCA, (**b**) W1.0-NCA.

To investigate the electrochemical performance of NCA and W1.0-NCA, cyclic voltammetry during the third initial cycle was performed at a scanning rate of 0.1 mV s^−1^, shown in Figure 6. Three pairs of redox peaks corresponding to phase transitions from H1 to M (hexagonal to monoclinic), M to H2 (monoclinic to hexagonal), and H2 to H3 (hexagonal to another hexagonal) phase can be observed during the lithium-ion deintercalation/intercalation process. Concretely, the redox potential difference (ΔV) is smaller for the W1.0-NCA (0.016 V) as compared to the value of NCA (0.047 V), suggesting a lower polarization and improved reversibility [42,43]. Furthermore, the phase transition from H2 to H3 (at about 4.2 V) is the main reason for the abrupt change of anisotropic volume. The peak intensity of H2 to H3 phase transition for NCA reduces in subsequent cycles as illustrated in Figure 6a, indicating the NCA sustained irreversible structural damage resulting from the anisotropic volume changes [17]. While for W1.0-NCA, shown in Figure 6b, this peak is well-overlapped, proving the reversibility of the H2 to H3 phase during cycling. Therefore, W1.0-NCA reveals lower polarization which maintains the reversibility of the H2 to H3 phase and improves the reaction kinetics, thus stabilizing the layered structure.

**Figure 7:**
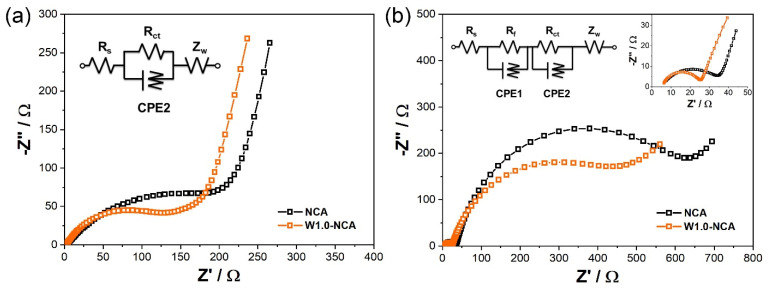
EIS and equivalent-circuit diagram of EIS curves fitting of NCA and W1.0-NCA during (**a**) before cycling and (**b**) after 100 cycles.

To further explore the electrochemical kinetics process of the NCA and W1.0-NCA, electrochemical impedance spectra (EIS) were performed. The Nyquist plots of NCA and W1.0-NCA before and after 100 cycles are displayed in Figure 7. Each Nyquist plot consists of semicircles in high to medium frequency region and a linear Warburg part at low frequency. The high-frequency semicircles represent the solid electrolyte interface layer impedance (R_f_) and the ohmic resistance of electrolytes (R_s_). The semicircles in the medium-frequency region are associated with charge transfer resistance (R_ct_), and slanted lines in the low-frequency region are related to Li^+^ diffusion in the solid electrodes (Z_w_). The equivalent circuit is given in Figure 7, and the relevant fitting results are presented in Table 2. After 100 cycles, the change in R_f_ and R_s_ is considerably smaller compared to the R_ct_ values, indicating that the impedance of the cell is dominated by the R_ct_. For the W1.0-NCA cathode, R_ct_ slightly changed after 100 cyclings (from 111.1 to 344.5 Ω), in contrast to the NCA (from 209.0 to 629.9 Ω). The results suggest that the W-doping greatly reduces the impedance of the W1.0-NCA cathodes, which facilitates the lithium-ion transfer during the charge-discharge process, while NCA suffered from a higher R_ct_ during the whole cycle.

**Figure 8:**
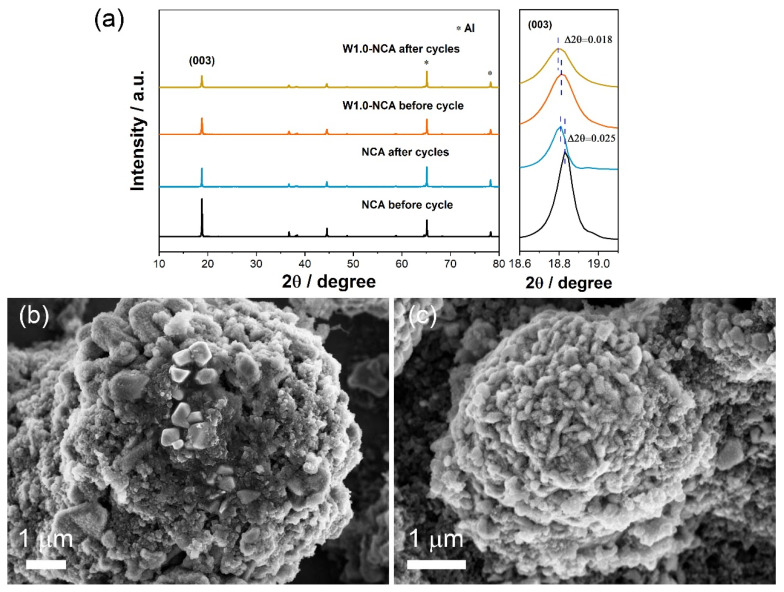
(**a**) XRD patterns of NCA and w1.0-NCA before and after 100 cycles, and the homologous magnified diffraction peaks (003). SEM images after 100 cycles of (**b**) NCA and (**c**) W1.0-NCA.

XRD patterns of NCA and W1.0-NCA before cycling and after 100 cycles were performed to investigate the structural stability. The materials were characterized without being removed from the aluminum foil. As demonstrated in Figure 8a, after 100 cycles at 1 C, the (003) peak of the NCA and W1.0-NCA both move to a lower position. Notably, the displacement of the (003) peak is 0.025° for NCA, while it was only 0.018° for W1.0-NCA. The results confirmed that W-doping could effectively improve cycle performance and stabilize the material structure [44]. Furthermore, Figure 8b,c shows the SEM images of NCA and W1.0-NCA cathode particles after 100 cycles. There were many microcracks in the spherical NCA after 100 cycles, and almost no microcracks were found in the W1.0-NCA cathode materials. Due to repeated volume changes, the original NCA particles are severely broken, which intensifies the side reaction with the electrolyte, and further leads to a significant increase in electrode impedance and rapid capacity attenuation. Therefore, W-doping effectively improves the interface stability and structural reversibility of the cathode materials.

## 4. Conclusions

W-doped Ni-rich NCA cathodes with smaller primary particle sizes were prepared via introducing WO_3_ during the sintering process. It was clearly shown that the 1.0 mol% W-doping of the NCA cathodes greatly enhanced their cycle stability as the W1.0-NCA cathodes retained 95.2% of their initial capacity after 100 cycles, compared to 86.3% for the NCA cathodes. The improvement of cycling stability by doping with W was primarily related to the stability of the bulk structure due to the reduction in internal deformations caused by the H2-H3 phase transformation, which prevented the production of microcracks. Moreover, the impedance spectroscopy analysis indicated that the W-doping suppressed sharp increase the charge transfer resistance. Taking all these factors into consideration, the excellent cycle performance and enhanced rate capability of W1.0-NCA cathodes make them promising for the high-performance LIB, which requires not only high energy density but also lower costs.

## Figures and Tables

**Table 1:** Rietveld refinement of XRD data.

Sample	a/Å	c/Å	c/a	Volume	I_(003)_/I_(104)_	Ni in Li/%	R_wp_/%	R/%
NCA	2.8709	14.1879	4.9420	101.268	1.62	3.09	1.495	0.928
W0.5-NCA	2.8718	14.1926	4.9421	101.365	1.49	4.14	1.493	0.925
W1.0-NCA	2.8721	14.1917	4.9412	101.384	1.38	6.36	1.463	1.038

**Table 2:** The fitted values of experimental data for NCA and W1.0-NCA.

Sample	Before Cycling		After 100 Cycles		
	R_s_/Ω	R_ct_/Ω	R_s_/Ω	R_f_/Ω	R_ct_/Ω
NCA	3.0	209.0	4.9	31.8	629.9
W1.0-NCA	2.3	111.1	5.4	20.3	344.5

## Data Availability

The data presented in this study are available from the corresponding author upon request.

## Supplementary Materials

The following supporting information can be downloaded at: https://www.mdpi.com/article/10.3390/nano12050729/s1, Figure S1: (a,b) Scanning electron microscopy (SEM) images of Ni_0.91_Co_0.09_(OH)_2_. (c) Element mapping of Ni, Co, and O for the Ni_0.91_Co_0.09_(OH)_2_ particle, Figure S2: Powder X-ray diffraction (XRD) patterns for Ni_0.91_Co_0.09_(OH)_2_, Figure S3: Rietveld refinement analysis of (a) NCA, (b) W0.5-NCA, and (c) W1.0-NCA. Figure S4: (a) X-ray photoelectron spectroscopy (XPS) survey spectrum of NCA, W0.5-NCA, and W1.0-NCA. (b) magnified view in the binding energy of 0–60 eV.

## References

[1] Liu T.C., Yu L., Liu J.J., Lu J., Bi X.X., Dai A., Li M., Li M.F., Hu Z.X., Ma L. (2021). Understanding Co roles towards developing Co-free Ni-rich cathodes for rechargeable batteries. Nat. Energy.

[2] Yu Z.L., Qu X.Y., Dou A.C., Zhou Y., Su M.R., Liu Y.J. (2021). Carbon-coated cation-disordered rocksalt-type transition metal oxide composites for high energy Li-ion batteries. Ceram. Int..

[3] Wang X.X., Ding Y.L., Deng Y.P., Chen Z.W. (2020). Ni-rich/Co-poor layered cathode for automotive Li-ion batteries: Promises and challenges. Adv. Energy Mater..

[4] Chen Z., Guo F.A., Zhang Y.X. (2021). Micron-sized monodisperse particle LiNi_0.6_Co_0.2_Mn_0.2_O_2_ derived by oxalate solvothermal process combined with calcination as cathode material for lithium-ion batteries. Materials.

[5] Salgado R.M., Danzi F., Oliveira J.E., El-Azab A., Camanho P.P., Braga M.H. (2021). The latest trends in electric vehicles batteries. Molecules.

[6] Chu B.B., You L.Z., Li G.X., Huang T., Yu A.S. (2021). Revealing the role of w-doping in enhancing the electrochemical performance of the LiNi_0.6_Co_0.2_Mn_0.2_O_2_ cathode at 4.5 V. ACS Appl. Mater. Interfaces.

[7] Goonetilleke D., Shaima N., Pang W.K., Peterson V.K., Petibon R., Li J., Dahn J.R. (2019). Structural evolution and high-voltage structural stability of Li(Ni_x_Mn_y_Co_z_)O_2_ electrodes. Chem. Mater..

[8] Kim U.H., Ryu H.H., Kim J.H., Mücke R., Kaghazchi P., Yoon C.S., Sun Y.K. (2019). Microstructure-controlled Ni-rich cathode material by microscale compositional partition for next-generation electric vehicles. Adv. Energy Mater..

[9] Zhang X.Y., Zhang P.P., Zeng T.Y., Yu Z.L., Qu X.Y., Peng X.Q., Zhou Y., Duan X.G., Dou A., Su M.R. (2021). Improving the structure stability of LiNi_0.8_Co_0.15_Al_0.05_O_2_ by double modification of tantalum surface coating and doping. ACS Appl. Energy Mater..

[10] Liu S.W., Li Y.J., Wang S.L., Chen Y.X., Tan Z.L., Yang J.C., Deng S.Y., He Z.J., Li C.X. (2021). Towards superior cyclability of LiNi_0.8_Co_0.15_Al_0.05_O_2_ cathode material for lithium-ion batteries via yttrium modification. J. Alloy. Compd..

[11] Schmuch R., Wagner R., Hörpel G., Placke T., Winter M. (2018). Performance and cost of materials for lithium-based rechargeable automotive batteries. Nat. Energy.

[12] Song C.H., Wang W.G., Peng H.L., Wang Y., Zhao C.L., Zhang H.B., Tang Q.W., Lv J.Z., Du X.J., Dou Y.M. (2018). Improving the electrochemical performance of LiNi_0.80_Co_0.15_Al_0.05_O_2_ in lithium-ion batteries by LiAlO_2_ surface modification. Appl. Sci..

[13] Qiu Z.P., Liu Z., Fu X.J., Liu J.M., Zeng Q.G. (2019). Improving the cycling performance of LiNi_0.80_Co_0.15_Al_0.05_O_2_ cathode materials via zirconium and fluorine co-substitution. J. Alloy. Compd..

[14] Park K.J., Choi M.J., Maglia F., Kim S.J., Kim K.H., Yoon C.S., Sun Y.K. (2018). High-capacity concentration gradient Li[Ni_0.865_Co_0.120_Al_0.015_]O_2_ cathode for lithium-ion batteries. Adv. Energy Mater..

[15] Qiu Z.P., Zhang Y.L., Liu Z., Gao Y., Liu J.M., Zeng Q.G. (2020). Stabilizing Ni-rich LiNi_0.92_Co_0.06_Al_0.02_O_2_ cathodes by boracic polyanion and tungsten cation co-doping for high-energy lithium-ion batteries. ChemElectroChem.

[16] Nie Y., Xiao W., Miao C., Wang J.L., Tan Y., Xu M.B., Wang C.J. (2021). Improving the structural stability of Ni-rich LiNi_0.81_Co_0.15_Al_0.04_O_2_ cathode materials with optimal content of trivalent Al ions doping for lithium ions batteries. Ceram. Int..

[17] Jamil S., Ran Q.W., Yang L., Huang Y., Gao S., Yang X.K., Wang X.Y. (2021). Improved high-voltage performance of LiNi_0.87_Co_0.1_Al_0.03_O_2_ by Li^+^-conductor coating. Chem. Eng. J..

[18] Zhang H.X., Yang S.Y., Huang Y.Y., Hou X.H. (2020). Synthesis of non-spherical LiNi_0.88_Co_0.09_Al_0.03_O_2_ cathode material for lithium-ion batteries. Energy Fuels.

[19] Xiong Y.K., Gao G.L., Li Y.J., Zhu J., Zheng J.C., Tan Z.L., Xi X.M., Yang J.C. (2021). The synergistic effect of Gd modification on improving the electrochemical performance of LiNi_0.88_Co_0.09_Al_0.03_O_2_ Cathode Materials. J. Electrochem. Soc..

[20] Yang H.P., Wu H.H., Ge M.Y., Li L.J., Yuan Y.F., Yao Q., Chen J., Xia L.F., Zheng J.M., Chen Z.Y. (2019). Simultaneously dual modification of Ni-rich layered oxide cathode for high-energy lithium-ion batteries. Adv. Funct. Mater..

[21] Kim U.H., Park G.T., Son B.K., Nam G.W., Liu J., Kuo L.Y., Kaghazchi P., Yoon C.S., Sun Y.K. (2020). Heuristic solution for achieving long-term cycle stability for Ni-rich layered cathodes at full depth of discharge. Nat. Energy.

[22] Liu L.H., Li M.C., Chu L.H., Jiang B., Lin R.X., Zhu X.P., Cao G.Z. (2020). Layered ternary metal oxides: Performance degradation mechanisms as cathodes, and design strategies for high-performance batteries. Prog. Mater. Sci..

[23] Li H.Y., Li J., Zaker N., Zhang N., Botton G.A., Dahn J.R. (2019). Synthesis of single crystal LiNi_0.88_Co_0.09_Al_0.03_O_2_ with a two-step lithiation method. J. Electrochem. Soc..

[24] Wu F., Liu N., Chen L., Su Y.F., Tan G.Q., Bao L.Y., Zhang Q.Y., Lu Y., Wang J., Chen S. (2019). Improving the reversibility of the H2-H3 phase transitions for layered Ni-rich oxide cathode towards retarded structural transition and enhanced cycle stability. Nano Energy.

[25] Ryu H.H., Park K.J., Yoon C.S., Sun Y.K. (2018). Capacity fading of Ni-rich Li[Ni_x_Co_y_Mn_1-x-y_]O_2_ (0.6 ≤ x ≤ 0.95) cathodes for high-energy-density lithium-ion batteries: Bulk or surface degradation?. Chem. Mater..

[26] Nam G.W., Park N.Y., Park K.J., Yang J.H., Liu J., Yoon C.S., Sun Y.K. (2019). Capacity fading of Ni-rich NCA cathodes: Effect of microcracking extent. ACS Energy Lett..

[27] Liu Y.L., Ouyang D.X., Rathore D., Wu H.H., Li K., Wang Y.Q., Sha J., Yin S., Dahn J.R. (2021). An evaluation of a systematic series of cobalt-free Ni-rich core-shell materials as positive electrode materials for Li-ion batteries. J. Electrochem. Soc..

[28] Wu K., Jiao J.Y., Li N., Wang M., Jia G.F., Lee Y.L., Dang R.B., Deng X., Xiao X.L., Wu Z.J. (2021). Revealing the multiple influences of Zr substitution on the structural and electrochemical behavior of high nickel LiNi_0.8_Co_0.1_Mn_0.1_O_2_ cathode material. J. Phys. Chem. C.

[29] Chang B., Kim J., Cho Y., Hwang I., Jung M.S., Char K., Li K.T., Kim K.J., Choi J.W. (2020). Highly elastic binder for improved cyclability of nickel-rich layered cathode materials in lithium-ion batteries. Adv. Energy Mater..

[30] Tian R.Z., Su J.R., Ma Z.J., Song D.W., Shi X.X., Zhang H.Z., Li C.L., Zhang L.Q. (2020). Influences of surface Al concentration on the structure and electrochemical performance of core-shell LiNi_0.8_Co_0.15_Al_0.05_O_2_ cathode material. Electrochim. Acta.

[31] Lu Y., Zhang Y.D., Zhang Q., Cheng F.Y., Chen J. (2020). Recent advances in Ni-rich layered oxide particle materials for lithium-ion batteries. Particuology.

[32] Choi C.M., Park J.H., Sun Y.K., Yoon C.S. (2021). Ultra-stable cycling of multi-doped (Zr, B) Li[Ni_0.885_Co_0.100_Al_0.015_]O_2_ cathode. J. Power Sources.

[33] Bai X., Wei A.J., He R., Li W., Li X.H., Zhang L.H., Liu Z.F. (2020). The structural and electrochemical performance of Mg-doped LiNi_0.85_Co_0.10_Al_0.05_O prepared by a solid state method. J. Electroanal. Chem..

[34] Li W.J., Zhuang W.D., Gao M., Zhou Y.N., Zhang J., Li N., Liu X.H., Huang W., Lu S.G. (2020). New insight into the role of Mn doping on the bulk structure stability and interfacial stability of Ni-rich layered oxide. ChemNanoMat.

[35] Zhou K., Xie Q., Li B.H., Manthiram A. (2021). An in-depth understanding of the effect of aluminum doping in high-nickel cathodes for lithium-ion batteries. Energy Storage Mater..

[36] Wang Y.Y., Sun Y.Y., Liu S., Li G.R., Gao X.P. (2018). Na-doped LiNi_0.8_Co_0.15_Al_0.05_O_2_ with excellent stability of both capacity and potential as cathode materials for Li-Ion batteries. ACS Appl. Energy Mater..

[37] Ryu H.H., Park G.T., Yoon C.S., Sun Y.K. (2019). Suppressing detrimental phase transitions via tungsten doping of LiNiO_2_ cathode for next-generation lithium-ion batteries. J. Mater. Chem. A.

[38] Kim U.H., Park N.Y., Park G.T., Kim H., Yoon C.S., Sun Y.K. (2020). High-energy W-doped Li[Ni_0.95_Co_0.04_Al_0.01_]O_2_ cathodes for next-generation electric vehicles. Energy Storage Mater..

[39] Li X.Q., Zhou L.M., Wang H., Meng D.C., Qian G.N., Wang Y., He Y.S., Wu Y.J., Hong Z.J., Ma Z.F. (2021). Dopants modulate crystal growth in molten salts enabled by surface energy tuning. J. Mater. Chem. A.

[40] Shang G.Z., Tang Y.W., Lai Y.Q., Wu J., Yang X., Li H.X., Peng C., Zheng J.F., Zhang Z. (2019). Enhancing structural stability unto 4.5 V of Ni-rich cathodes by tungsten-doping for lithium storage. J. Power Sources.

[41] Che W., Wan X.W., Zhang D.Y., Chang C.K. (2021). Stabilized performance of LiNi_0.90_Co_0.07_Al_0.03_O_2_ cathodes via Zr^4+^ doping upon 4.5 V application due to the suppression of H2-H3 Phase Transitions. ACS Sustain. Chem. Eng..

[42] He H.H., Dong J., Zhang D.Y., Chang C.K. (2020). Effect of Nb doping on the behavior of NCA cathode: Enhanced electrochemical performances from improved lattice stability towards 4.5V application. Ceram. Int..

[43] Gan Z.G., Hu G.R., Peng Z.D., Cao Y.B., Tong H., Du K. (2019). Surface modification of LiNi_0.8_Co_0.1_Mn_0.1_O_2_ by WO_3_ as a cathode material for LIB. Appl. Surf. Sci..

[44] Guo F.Y., Xie Y.F., Zhang Y.X. (2021). Low-temperature strategy to synthesize single-crystal LiNi_0.8_Co_0.1_Mn_0.1_O_2_ with enhanced cycling performances as cathode material for lithium-ion batteries. Nano Res..

