# Supplementary material for: Enhancing the Electrochemical Performance of Ni-Rich LiNi0.88Co0.09Al0.03O2 Cathodes through Tungsten-Doping for Lithium-Ion Batteries
Source: Nanomaterials (Basel). 2022 Feb 22;12(5):729. doi: 10.3390/nano12050729 (PMC8912114; doi:10.3390/nano12050729)
Supplement: Supplementary file 1 [file nanomaterials-12-00729-s001.zip › nanomaterials-1603245-supplementary.pdf]

Supporting Information

Enhancing the Electrochemical Performance of Ni-Rich $LiNi_{0.88}Co_{0.09}Al_{0.03}O_2$ Cathodes Through Tungsten-Doping for Lithium-Ion Batteries

Rui Zhang, Hengrui Qiu and Youxiang Zhang *

College of Chemistry and Molecular Sciences, Wuhan University, Wuhan 430072, China; zhangrui@whu.edu.cn (R.Z.); 2020102030003@whu.edu.cn (H.Q.)

* Correspondence: yxzhang04@whu.edu.cn

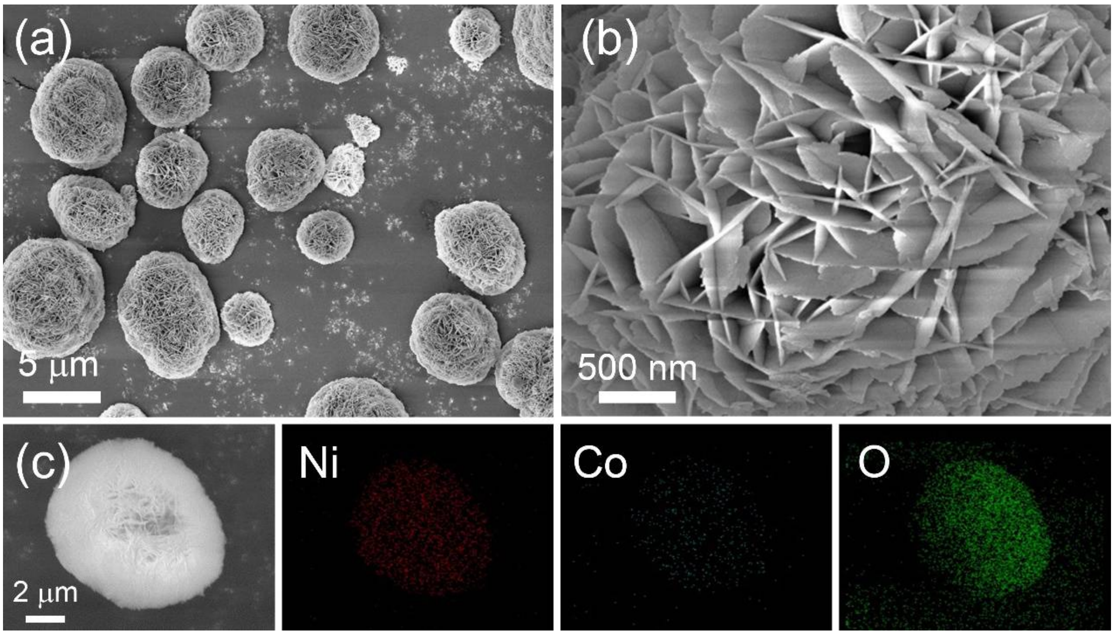

Figure S1. (**a,b**) Scanning electron microscopy (SEM) images of $Ni_{0.91}Co_{0.09}(OH)_2$. (**c**) Element mapping of Ni, Co and O for the $Ni_{0.91}Co_{0.09}(OH)_2$ particle.

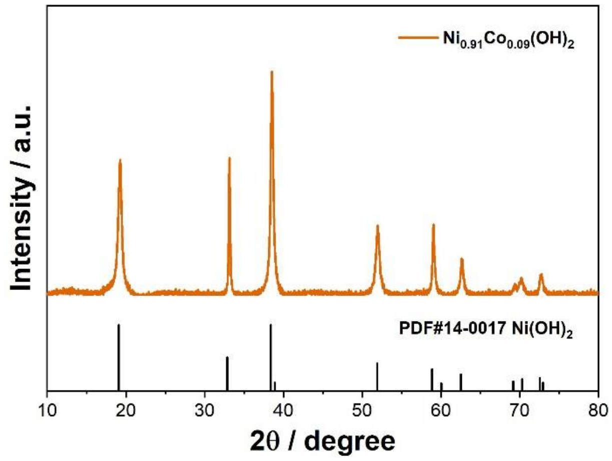

Figure S2. Powder X-ray diffraction (XRD) patterns for $Ni_{0.91}Co_{0.09}(OH)_2$.

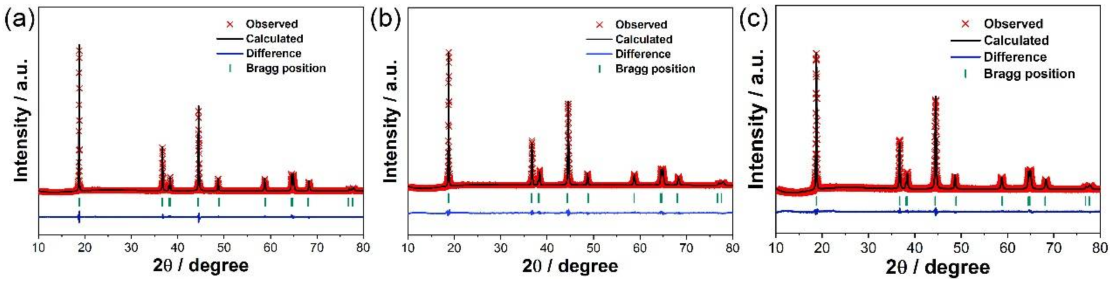

Figure S3. Rietveld refinement analysis of (**a**) NCA, (**b**) W0.5-NCA, and (**c**) W1.0-NCA.

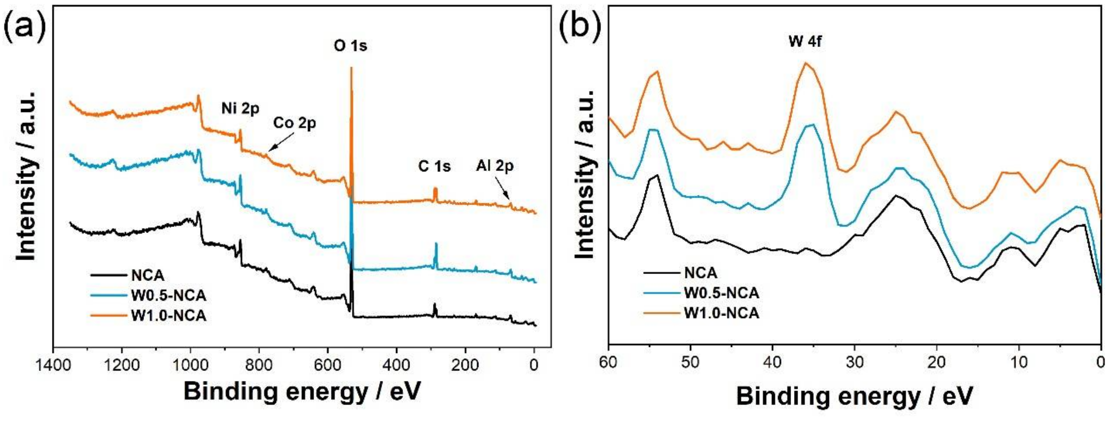

Figure S4. (**a**) X-ray photoelectron spectroscopy (XPS) survey spectrum of NCA, W0.5-NCA, and W1.0-NCA. (**b**) magnified view in the binding energy of 0–60 eV.
